# High prevalence of long COVID in anti-TPO positive euthyroid individuals with strongly elevated SARS-CoV-2-specific T cell responses and moderately raised anti-spike IgG levels 23 months post-infection

**DOI:** 10.3389/fimmu.2024.1448659

**Published:** 2024-10-10

**Authors:** Zsolt Matula, Viktória Király, Gabriella Bekő, Márton Gönczi, András Zóka, Róbert Steinhauser, Ferenc Uher, István Vályi-Nagy

**Affiliations:** ^1^ Laboratory for Experimental Cell Therapy, Central Hospital of Southern Pest, National Institute of Hematology and Infectious Diseases, Budapest, Hungary; ^2^ Central Laboratory of Central Hospital of Southern Pest, National Institute of Hematology and Infectious Diseases, Budapest, Hungary; ^3^ Department of Hematology and Stem Cell Transplantation, Central Hospital of Southern Pest, National Institute of Hematology and Infectious Diseases, Budapest, Hungary

**Keywords:** SARS-CoV-2, long covid, long-term immunity, autoantibody characterization, anti-TPO, anti-spike IgG, T cell responses, IFNγ ELISpot assay

## Abstract

**Introduction:**

Severe acute respiratory syndrome coronavirus type 2 (SARS-CoV-2) infection, the causative agent of coronavirus disease 2019 (COVID-19), causes post-acute infection syndrome in a surprisingly large number of cases worldwide. This condition, also known as long COVID or post-acute sequelae of COVID-19, is characterized by extremely complex symptoms and pathology. There is a growing consensus that this condition is a consequence of virus-induced immune activation and the inflammatory cascade, with its prolonged duration caused by a persistent virus reservoir.

**Methods:**

In this cross-sectional study, we analyzed the SARS-CoV-2-specific T cell response against the spike, nucleocapsid, and membrane proteins, as well as the levels of spike-specific IgG antibodies in 51 healthcare workers, categorized into long COVID or convalescent control groups based on the presence or absence of post-acute symptoms. Additionally, we compared the levels of autoantibodies previously identified during acute or critical COVID-19, including anti-dsDNA, anti-cardiolipin, anti-β2-glycoprotein I, anti-neutrophil cytoplasmic antibodies, and anti-thyroid peroxidase (anti-TPO). Furthermore, we analyzed the antibody levels targeting six nuclear antigens within the ENA-6 S panel, as positivity for certain anti-nuclear antibodies has recently been shown to associate not only with acute COVID-19 but also with long COVID. Finally, we examined the frequency of diabetes in both groups. Our investigations were conducted at an average of 18.2 months (convalescent control group) and 23.1 months (long COVID group) after confirmed acute COVID-19 infection, and an average of 21 months after booster vaccination.

**Results:**

Our results showed significant differences between the two groups regarding the occurrence of acute infection relative to administering the individual vaccine doses, the frequency of acute symptoms, and the T cell response against all structural SARS-CoV-2 proteins. A statistical association was observed between the incidence of long COVID symptoms and highly elevated anti-TPO antibodies based on Pearson's chi-squared test. Although patients with long COVID showed moderately elevated anti-SARS-CoV-2 spike IgG serum antibody levels compared to control participants, and further differences were found regarding the positivity for anti-nuclear antibodies, anti-dsDNA, and HbA1c levels between the two groups, these differences were not statistically significant.

**Disscussion:**

This study highlights the need for close monitoring of long COVID development in patients with elevated anti-TPO titers, which can be indicated by strongly elevated SARS-CoV-2-specific T cell response and moderately raised anti-spike IgG levels even long after the acute infection. However, our results do not exclude the possibility of new-onset thyroid autoimmunity after COVID-19, and further investigations are required to clarify the etiological link between highly elevated anti-TPO titers and long COVID.

## Introduction

In contrast to those who recovered from coronavirus infection without complications, many patients suffer worldwide from the post-acute sequelae of COVID-19 (PASC). Even conservative estimates suggest that approximately 10% of patients experience the long-term consequences of COVID-19, posing a new challenge to healthcare systems (1). The World Health Organization defines PASC or long COVID as the persistence of symptoms or the emergence of new symptoms lasting more than two months after SARS-CoV-2 infection (2). For some individuals, symptoms characteristic of the acute phase fail to resolve or diminish, leading to persistent symptoms, whereas others experience new symptoms that have not previously occurred. The severity of these symptoms varies widely, ranging from mild symptoms, such as fatigue, weakness, and general malaise, to more severe conditions causing incapacitation, such as attention deficits, memory complaints, shortness of breath, chest pain, heart issues, various neurological disorders, or severe dysautonomia (3). In the case of long COVID, we are dealing with an extremely diverse clinical condition, as more than 200 PASC symptoms have been reported, affecting multiple organ systems, including the cardiovascular, respiratory, nervous, integumentary, musculoskeletal, digestive, endocrine, urinary, and even the reproductive systems (4). Therefore, in recent years, a vast amount of research has been conducted worldwide to define the pathogenesis of long COVID, which has helped to understand various aspects of this highly complex condition. However, even today, it is impossible to establish clear cause-and-effect relationships regarding the development and diverse courses of long COVID.

There is a high degree of consensus in the literature that a persistent SARS-CoV-2 reservoir can contribute to the pathology of long COVID through several mechanisms, including endothelial activation and coagulation, dysbiosis, neuroimmune dysregulation, latent virus reactivation such as EBV, and systemic immune dysregulation (5–7). Additionally, the SARS-CoV-2 reservoir may alter vagal nerve signaling, causing nonspecific long COVID symptoms such as fatigue, impaired concentration, muscle and joint pain, sleep disturbances, inappetence, anxiety, depression, and autonomic dysfunction (8, 9). Furthermore, it can induce cognitive, neurological, and psychiatric symptoms through neuroinvasion and nerve inflammation (10, 11). Direct evidence for the existence of the virus reservoir is the identification of SARS-CoV-2-specific proteins or RNA in plasma or tissue biopsies (12–16). However, we can also infer the presence of SARS-CoV-2 reservoirs indirectly based on the adaptive immune response. However, studies on this matter have been contradictory. While some groups have detected a higher proportion of functionally responsive T cells (17, 18) and higher serum antibody levels in individuals with post-acute symptoms (19), other groups have not observed any differences in antibody and T cell responses (20) or reported lower antibody levels and similar SARS-CoV-2-specific T cell responses to those of the convalescent control group (21). It is becoming increasingly evident that long-term systemic inflammation, T cell dysregulation, and CD8+ T cell exhaustion occur in patients with long COVID (19).

There is substantial evidence that SARS-CoV-2 infection contributes to the development of cross-reactive autoimmunity, affecting numerous self-antigens in the central nervous system (22, 23), skeletal and smooth muscles, joints, skin, lungs, kidneys, heart, blood vessels, and many other tissues (24–26). During the early period of the pandemic, a large and diverse repertoire of autoantibodies (AABs) was identified that are induced throughout acute or critical COVID-19. These include rheumatoid factor, anti-nuclear antibodies such as anti-Ro/SSA, anti-La/SSB, anti-RNP, anti-histone, and anti-dsDNA, as well as anti-neutrophilic cytoplasmic antibodies (ANCA), anti-Saccharomyces cerevisiae antibodies (ASCA) (24, 27–32), and anti-phospholipid antibodies such as lupus anticoagulant, anti-cardiolipin, and anti-β2 glycoprotein I (33–35), among many others. Since the clinical characteristics of autoimmunity associated with COVID-19 show a high degree of overlap with post-recovery short- or medium-term autoimmune parameters, and even with features of ‘classic’ autoimmune diseases such as systemic lupus erythematosus, rheumatoid arthritis, vasculitis, and antiphospholipid syndrome, it is a significant challenge to identify AABs that are highly specific to long COVID rather than acute COVID-19 or other autoimmune diseases. So far, only a few studies suggest a clear correlation between certain AABs and long COVID (27, 36–40).

In this cross-sectional study, we determined the spike-, nucleocapsid-, and membrane-specific T cell responses in parallel with the spike-specific IgG antibody levels in long COVID patients and convalescent control individuals more than 20 months after acute infection. Additionally, we compared T cell responses against SARS-CoV-2 epitopes with a high degree of homology with endemic coronaviruses between the two groups. We evaluated the frequency of acute symptoms, occurrence of acute infections relative to administering the individual vaccine doses in the two groups, and frequency of the most common post-acute symptoms in the long COVID group. The demographic distribution (age and sex) of the participants, the type of the three vaccine doses they received, and the time of administration were very similar since the subjects of this study were all healthcare workers. Furthermore, we determined the levels of several AABs previously identified in acute or critical COVID-19 cases, including anti-dsDNA, anti-cardiolipin, anti-β2-glycoprotein I, anti-neutrophil cytoplasmic antibodies, and anti-TPO. In addition, we assessed the levels of AABs with the ENA6S panel targeting six nuclear antigens: SS-B, SS-A 52 kDa, Scl 70, Jo-1, snRNP/Sm, Sm, and SS-A 60 kDa. To date, only a few studies have shown a causal relationship between long COVID phenotypes and the presence of these antibodies. Finally, we investigated the relationship between long COVID and diabetes.

## Materials and methods

### Study population

All participants were recruited based on voluntary application, and informed consent was obtained from each participant. The study was approved by the Ethics and Scientific Committee of Central Hospital of Southern Pest, National Institute of Hematology and Infectious Diseases (OGYÉI/50268-8/2017). The participants are all healthcare workers, the vast majority of whom received the first and second doses of the mRNA-based BNT162b2 vaccine (Pfizer-BioNTech) 21 days apart between January and March 2021, followed by a third (booster) dose of the BNT162b2 vaccine 6-9 months later, between September and November 2021. A small proportion of the participants were immunized with other heterologous vaccine regimens, utilizing the BBIBP-CorV (Sinopharm), Ad26.COV2.S (Janssen-Cilag), and Gam-COVID-Vac (Sputnik V) vaccines in addition to BNT162b2. All participants had confirmed SARS-CoV-2 infection by PCR or antigen test. Participants were recruited in April and May 2023, when acute and residual symptoms were summarized based on self-administered questionnaire surveys. Based on these surveys, participants were classified into the convalescent control or long COVID group. Individuals who reported at least two different post-acute symptoms that persisted for at least 3 months were classified into the Long COVID group, while participants in the convalescent control group did not report any residual symptoms in the post-recovery period. Our investigations were conducted between June and August 2023, with each participant providing a peripheral blood sample at a single time point. At that time, an average of 18.2 months (convalescent control group) and 23.1 months (long COVID group) had passed since confirmed acute COVID-19 infection, and an average of 21 months since booster vaccination. The detailed characteristics of individuals of both groups are shown in **Table 1**. All participants in this study were regularly screened for SARS-CoV-2 infection by PCR tests between September 1, 2020, and September 30, 2023. In case of symptoms suggestive of COVID-19, the participants were tested for SARS-CoV-2 infection as soon as possible. COVID-19 was confirmed by the Allplex SARS-CoV-2 PCR Assay (Seoul, South Korea) whenever possible or by the CLINITEST Rapid COVID-19 Antigen Test (SIEMENS Healthineers, Erlangen, Germany).

**Table 1:** Detailed characteristics of study participants.

	Convalescent control (CC) group	Long COVID (LC) group
Study participants	Vaccinated and infected individuals without persistent symptoms	Vaccinated and infected individuals with persistent symptoms
Number of individuals	27	24
Age (mean)	49	52
Age range	30-65	27-67
Gender		
Female Male	27 0	23 1
Vaccine combinations	BNT162b2+BNT162b2+BNT162b2: 87% BNT162b2+BNT162b2+BBIBP-CorV: 8% Other combinations: 5%	BNT162b2+BNT162b2+BNT162b2: 96% Other combinations: 4%
Time elapsed since the last known infection at sampling time (mean)	18.2 months	23.1 months
Duration of long COVID symptoms	–	3 months: 54% 6 months: 25% More than 6 months: 21%

### Determination of SARS-CoV-2-specific T cell response

The SARS-CoV-2-specific T cell response was assessed using the T-SPOT Discovery SARS-CoV-2 kit (Oxford Immunotec Ltd., Abingdon, UK) following the manufacturer’s instructions. Venous blood samples were collected in Vacuette 9NC Coagulation sodium citrate tubes (Greiner Bio-One, Kremsmünster, Austria), and peripheral blood mononuclear cells were isolated by density gradient centrifugation using a Leucosep Kit (Oxford Immunotec). A total of 250,000 viable cells were plated in each well of a 96-well microtiter plate provided in the kit. The cells were incubated for 18-20 hours with three different peptide pools specific to the spike (S), nucleocapsid (N), and membrane (M) proteins. A peptide pool specific to SARS-CoV-2 epitopes with a high degree of homology to endemic coronaviruses (Panel 13) was included in the analysis. The number of IFNγ-producing effector T cells was determined for all viral antigens using an AID vSPOT ELISPOT reader (AID Autoimmun Diagnostika, Strassberg, Germany) by counting the spots in each well. The number of spot-forming units (SFUs) per 250,000 PBMCs was calculated as the total number of T-spots for S, N, M, and Panel 13, minus the background for each well.

### Measurement of spike protein-specific (anti-S) IgG antibody levels

Venous blood samples were collected in Vacuette CAT Serum Separator Clot Activator tubes (Greiner Bio-One) and centrifuged for 10 minutes at 3500 RPM at room temperature. Anti-S IgG antibody levels were determined using the LIAISON SARS-CoV-2 TrimericS IgG indirect chemiluminescence immunoassay (CLIA) test kit (Diasorin, Saluggia, Italy) from serum samples, according to the manufacturer’s instructions. The light signal, based on a chemiluminescent reaction and proportional to the anti-S IgG antibodies present in the serum samples, calibrators, or controls, was measured using the Liaison XL chemiluminescence analyzer (Diasorin).

### Assessment of serum autoantibody levels

Venous blood samples were collected in Vacuette CAT Serum Separator Clot Activator tubes (Greiner Bio-One) and centrifuged for 10 minutes at 3500 RPM at room temperature. The AESKULISA ENA-6S solid-phase enzyme immunoassay (ELISA) was used for the combined qualitative determination of IgG AABs against six different nuclear antigens, including SS-B, SS-A 52 kDa, Scl 70, Jo-1, snRNP/Sm, Sm, and SS-A 60 kDa, in serum samples. The AESKULISA dsDNA-G solid-phase enzyme immunoassay with human recombinant double-stranded DNA (dsDNA) was assessed for quantitative and qualitative detection of IgG antibodies against dsDNA in serum samples. Serum samples from study participants were analyzed using the AESKULISA Cardiolipin-Check solid-phase enzyme immunoassay, which employs highly purified cardiolipin and native human ß2-glycoprotein I for the combined quantitative and qualitative detection of IgA, IgG, and IgM antibodies against cardiolipin. The AESKULISA β2-Glyco-Check solid-phase enzyme immunoassay, employing highly purified native β2-glycoprotein I from human plasma, was used for the combined quantitative and qualitative detection of IgA, IgG, and IgM antibodies against β2-glycoprotein I in serum samples. IMTEC-MPO-ANCA and IMTEC-PR3-ANCA indirect solid-phase enzyme immunoassays were used to quantitatively measure IgG class AABs against myeloperoxidase and proteinase 3, respectively, in serum samples. All measurements were performed according to the manufacturer’s instructions using an AP 22 ELITE fully automated ELISA system. The instrument automatically performed the ELISA tests, from sample pre-dilution, sample and reagent dispensing, incubation, and washing cycles to the final ELISA reading and data analysis. Finally, thyroid peroxidase antibody levels were determined in serum samples using a two-stage chemiluminescent microparticle immunoassay (CMIA) with the ARCHITECT Anti-TPO reagent kit (B2K47H). Specimens were analyzed using the Architect i2000SR chemiluminescent microparticle immunoassay analyzer (Abbott Diagnostics, Abbott Park, IL, USA) according to the manufacturer’s instructions.

### Measurement of serum TSH levels

Venous blood samples were collected in Vacuette CAT Serum Separator Clot Activator tubes (Greiner Bio-One) and centrifuged for 10 minutes at 3500 RPM at room temperature. TSH levels were quantified in the serum samples according to the manufacturer’s instructions by two-stage chemiluminescent microparticle immunoassay (CMIA) using the Architect TSH reagent kit (B7K62H). All specimens were analyzed using the Architect i2000SR chemiluminescent microparticle immunoassay analyzer (Abbott Diagnostics).

### Measurement of HbA1c levels

Venous blood samples were collected in Vacuette K3EDTA tubes (Greiner Bio-One). HbA1c levels were determined in EDTA-anticoagulated whole blood using an ADAMS A1c HA-8180V fully automated high-pressure liquid chromatography (HPLC) system (ARKRAY Inc., Kyoto, Japan). Column units, eluents, hemolyzing washing solutions, and calibrators were obtained from the manufacturer and the device was calibrated according to the manufacturer’s instructions.

### Statistical analysis

All statistical analyses comparing the T cell response, anti-S IgG response, and TSH, HbA1c, and anti-TPO levels between the long COVID and convalescent control groups were performed using a two-tailed t-probe and f-probe, and p-values of <0.05 were considered statistically significant (*p < 0.05; **p < 0.01). Pearson’s correlation analysis was performed to assess the correlation between T cell responses against endemic coronavirus-homolog epitopes and each structural SARS-CoV-2 protein (S, N, M). The relationship between long COVID symptoms and different AAB/TSH/HbA1c positivity was determined using Pearson’s chi-square test with Yates correction. Statistical analyses were performed using GraphPad Prism 6 software (version 6.07). Positive and negative cutoff values were determined based on the manufacturer’s package inserts.

## Results

### Vaccination schedule, sample collection, and occurrence of acute infection among study participants

**Figure 1:**
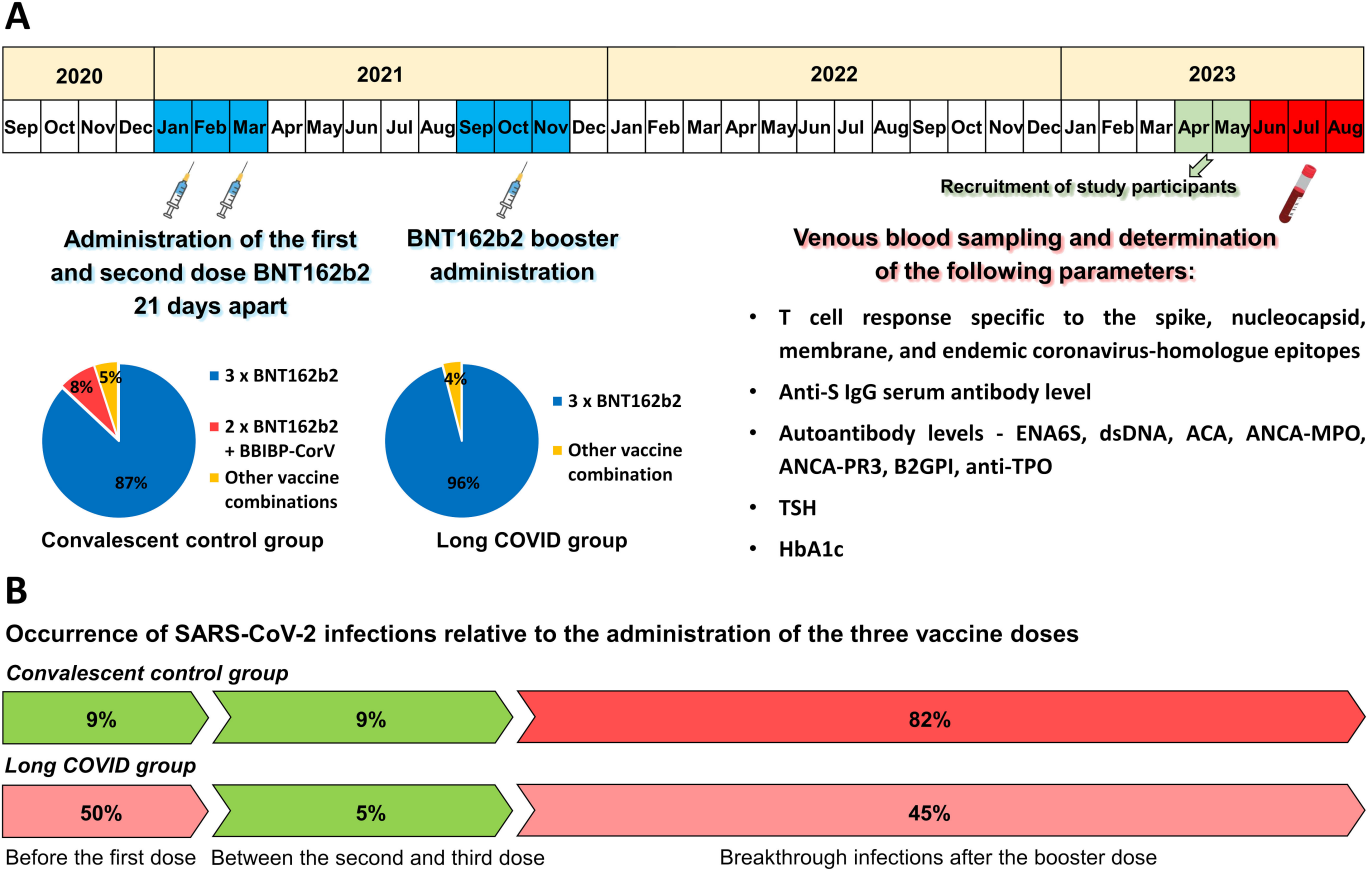
Vaccination timeline, sample collection, measurements conducted in this study, and the incidence of SARS-CoV-2 infections relative to vaccinations. **(A)** On average, participants received a third (booster) dose 7.4 months after the initial two-dose vaccine regimen. 96% of the long COVID group and 86% of the convalescent control group received three doses of the mRNA-based BNT162b2 vaccine. The remaining participants were immunized with other heterologous vaccine regimens. Samples were collected on average 21 months after the booster dose administration and were analyzed for spike-, nucleocapsid-, membrane-, and endemic coronavirus-specific T cell responses, serum anti-spike IgG levels, as well as several autoantibody levels, TSH, and HbA1c levels. **(B)** Significant differences were found between the two groups regarding the time point of acute infection relative to vaccinations. SARS-CoV-2 infections were confirmed by positive PCR or antigen tests. 82% of participants in the CC group were infected after receiving the third dose, with only 9% being infected before the first dose and 9% between the second and third doses. In contrast, 50% of long COVID patients were infected before receiving the first dose, only 5% between the second and third doses, and 45% after administering the third dose.

In this cross-sectional study, healthcare workers were divided into two groups depending on whether they had experienced post-acute sequelae of COVID-19 or not. Participants were recruited in April-May 2023 when acute and residual symptoms were summarized based on self-reported questionnaire surveys. Individuals in the convalescent control group (CC group) did not report any residual symptoms following recovery from COVID-19. In contrast, all participants in the long COVID group (LC group) reported at least two persistent or recurring post-acute symptoms lasting at least 3 months. Our investigations were conducted over a period from June to August 2023, with each participant providing a peripheral blood sample at one time point. 86% of the CC group and 96% of the LC group received the first and second doses of the mRNA-based BNT162b2 vaccine 21 days apart, during the period from January to March 2021. On average, 7.4 months after the second dose, between September and November 2021, all participants received a BNT162b2 booster. 13% of individuals in the CC group and 4% of patients in the LC group were immunized with other vaccination regimens, including the Ad26.COV2.S (Janssen-Cilag), Gam-COVID-Vac (Sputnik V), or BBIBP-CorV (Sinopharm) vaccines, in addition to the mRNA-based BNT162b2. At the study time, from June to August 2023, an average of 21 months had passed since the third (booster) dose was administered (**Figure 1A**). Of the 51 study participants, 50 were female, with an average age of 49 (CC group) and 52 years (LC group) at the time of study. All participants had symptomatic SARS-CoV-2 infection, confirmed mostly by a PCR test and in a smaller portion by an antigen test. Notably, we observed significant differences between the two groups regarding the occurrence of acute infection relative to administering the individual vaccine doses. The vast majority (82%) of the individuals in the CC group were infected after booster vaccination. In comparison, only 9-9% were infected before administering the first dose and between receiving the second and third doses, respectively (**Figure 1B**). In contrast, 50% of the participants in the LC group were infected before vaccination, and only 5% became ill between the second and third doses, while 45% were infected after the booster vaccination. Accordingly, the average time elapsed since the confirmed SARS-CoV-2 infection was 18.2 months in the CC group and 23.1 months in the LC group at the study time (June-August 2023).

### Frequency of COVID-19 and long COVID symptoms in the convalescent control and long COVID groups

**Figure 2:**
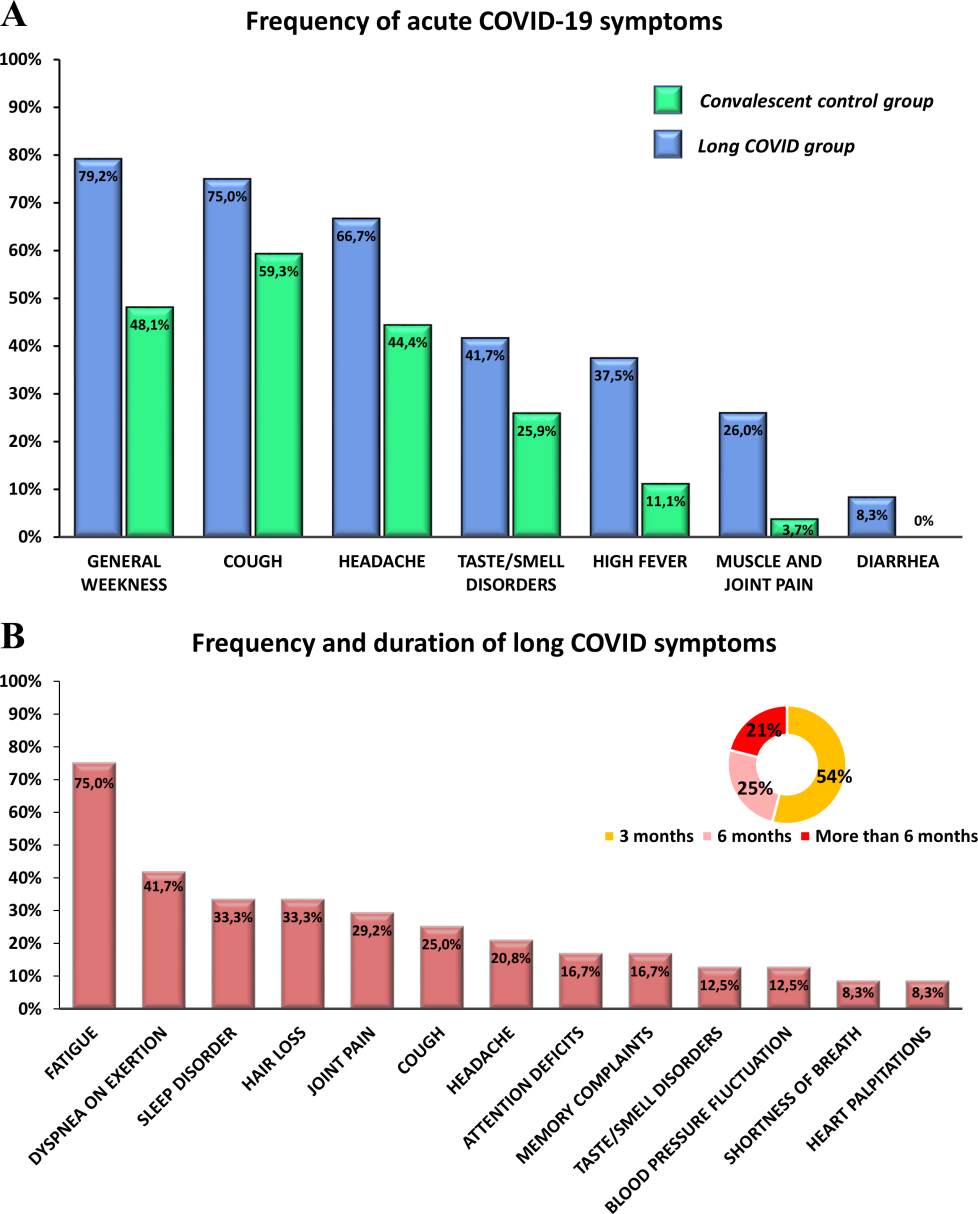
Frequency of different symptoms during acute COVID-19 in the convalescent control and long COVID groups, and the frequency and duration of post-acute symptoms in the long COVID group. **(A)** The prevalence of different symptoms during acute COVID-19 including general weakness, cough, headache, taste and smell disorders, high fever, muscle and joint pain, and diarrhea was compared between the long COVID and convalescent control groups. **(B)** The frequency and duration of post-acute symptoms such as fatigue, dyspnea on exertion, sleep disorders, hair loss, joint pain, cough, headache, attention deficit, memory complaints, taste and smell disorders, blood pressure fluctuations, shortness of breath, and palpitations, were assessed in the long COVID group.

Acute and long COVID symptoms were summarized based on self-reported questionnaire surveys. In general, acute symptoms such as general weakness, cough, headache, taste and smell disorders, high fever, muscle and joint pain, and diarrhea were typically more prevalent and emerged with greater severity in the LC group than in the CC group (**Figure 2A**). The most common post-acute symptoms in the LC group included fatigue, dyspnea on exertion, sleep disturbances, and hair loss. Additionally, participants reported persistent joint pain, cough, headache, attention deficit, memory complaints, taste and smell disorders, blood pressure fluctuations, shortness of breath, and palpitations. Long COVID symptoms persisted for an average of 3 months in 54%, 6 months in 25%, and more than 6 months in 21% of the participants (**Figure 2B**).

### Comparison of SARS-CoV-2-specific T cell responses and anti-SARS-CoV-2 spike IgG serum antibody levels between long COVID patients and convalescent control participants

**Figure 3:**
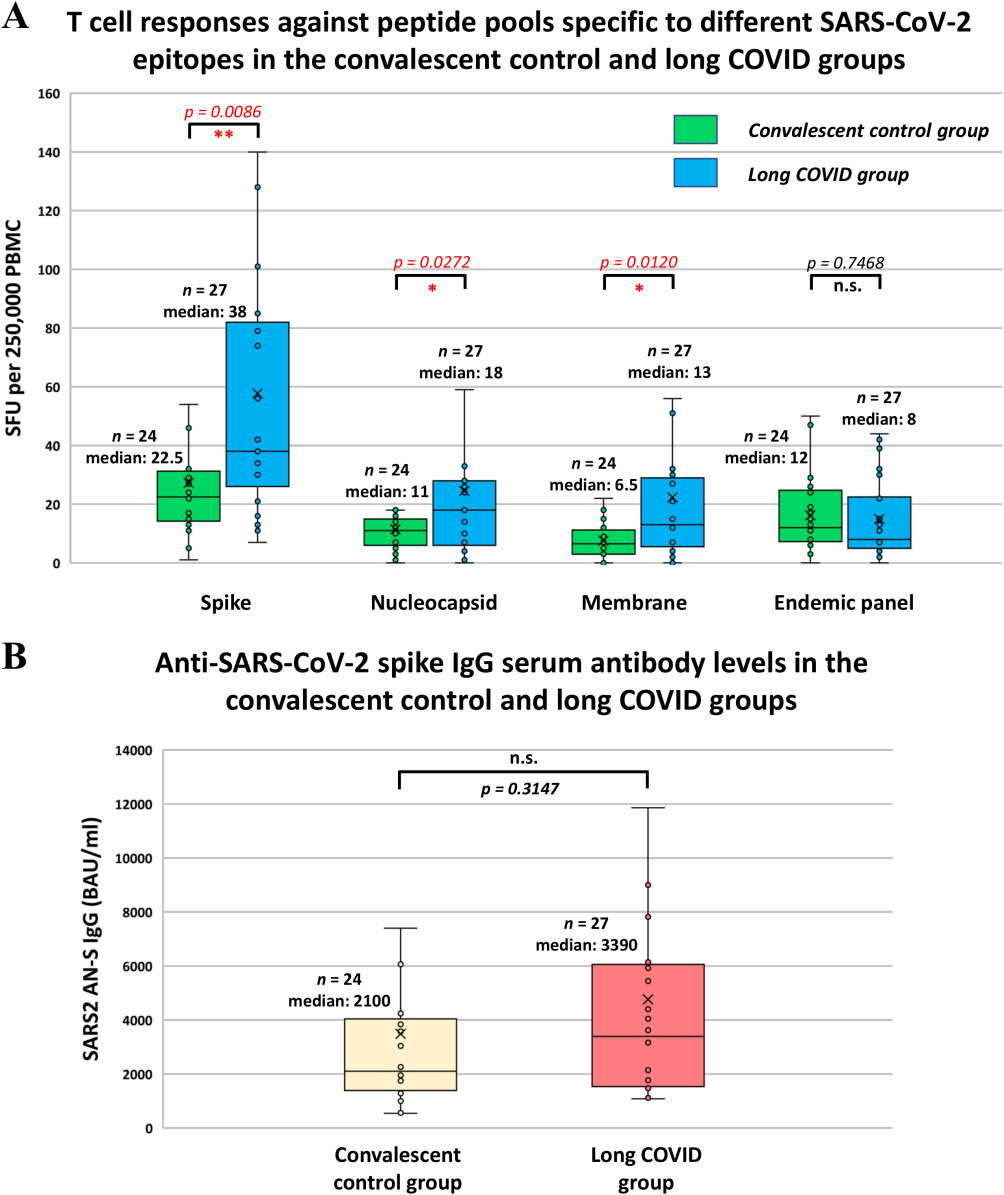
T cell responses against different SARS-CoV-2 epitopes and anti-SARS-CoV-2 spike IgG serum antibody levels in the convalescent control and long COVID groups. **(A)** T-SPOT Discovery SARS-CoV-2 ELISpot assay was employed to detect IFN-γ release from peripheral blood mononuclear cells after exposure to SARS-CoV-2 spike (S), nucleocapsid (N), membrane (M) peptides, as well as to endemic coronavirus-specific peptides. The number of spot-forming units (SFU) specific to S, N, M, and endemic coronavirus epitopes was determined as the total number of T-SPOTs for each peptide pool per 2.5 × 10^5^ PBMC minus the background. **(B)** The SARS-CoV-2-specific anti-S IgG antibody levels were determined by the by the LIAISON SARS-CoV-2 TrimericS IgG indirect chemiluminescence immunoassay (CLIA) test kit. **(A, B)** Statistical analyses utilized Student’s t-test, considering p-values of <0.05 as statistically significant (* p < 0.05; ** p < 0.01), whereas p-values of >0.05 were considered non-significant (n.s.). Box plots illustrate the median values with the interquartile range (lower and upper hinges) and ±1.5-fold of the interquartile range from the first and third quartiles (lower and upper whiskers).

The number of IFNγ-secreting virus-specific T cells was determined in both groups against each structural SARS-CoV-2 protein, including spike, nucleocapsid, and membrane proteins. In general, considerable variability was observed among individuals in both groups. When comparing the average SFU values measured in the two groups, a significantly higher frequency of SARS-CoV-2-specific T cells was found in the peripheral blood of long COVID patients compared to the convalescent control participants against the spike (medians of 22.5 SFU/2.5x10^5^ PBMC in the CC and 38 SFU/2.5x10^5^ PBMC in the LC group; *p* = 0.0086), nucleocapsid (medians of 11 SFU/2.5x10^5^ PBMC in the CC and 18 SFU/2.5x10^5^ PBMC in the LC group; *p* = 0.0272), and membrane protein (medians of 6.5 SFU/2.5x10^5^ PBMC in the CC and 13 SFU/2.5x10^5^ PBMC in the LC group; *p* = 0.012) (**Figure 3A**). However, we observed very similar T cell responses in both groups against SARS-CoV-2 epitopes showing a high degree of homology with endemic coronaviruses (medians of 12 SFU/2.5x10^5^ PBMC in the CC and 8 SFU/2.5x10^5^ PBMC in the LC group; *p* = 0.7468). Interestingly, Pearson’s correlation analysis revealed a strong positive correlation between T cell responses against individual structural SARS-CoV-2 viral proteins (S, N, M) and T cell responses against epitopes characteristic of endemic coronavirus strains in the LC group (**Supplementary Figure S1A**). However, in the CC group, no correlation was observed in this regard probably because of the high number of low SFU values (zero or near zero) against S, N, and M antigens (**Supplementary Figure S1B**). Similar to the T cell response, dramatic variabilities were observed between individuals within the CC and LC groups regarding anti-S IgG antibody levels. Although the median anti-S IgG antibody level of the LC group was notably, 1.6 times higher than that of the CC group, the difference was not statistically significant (medians of 2100 BAU/ml in the CC and 3390 BAU/ml in the LC group; *p* = 0.3147) (**Figure 3B**).

### Comparison of different autoantibody, TSH, and HbA1c levels between long COVID patients and convalescent control participants

**Figure 4:**
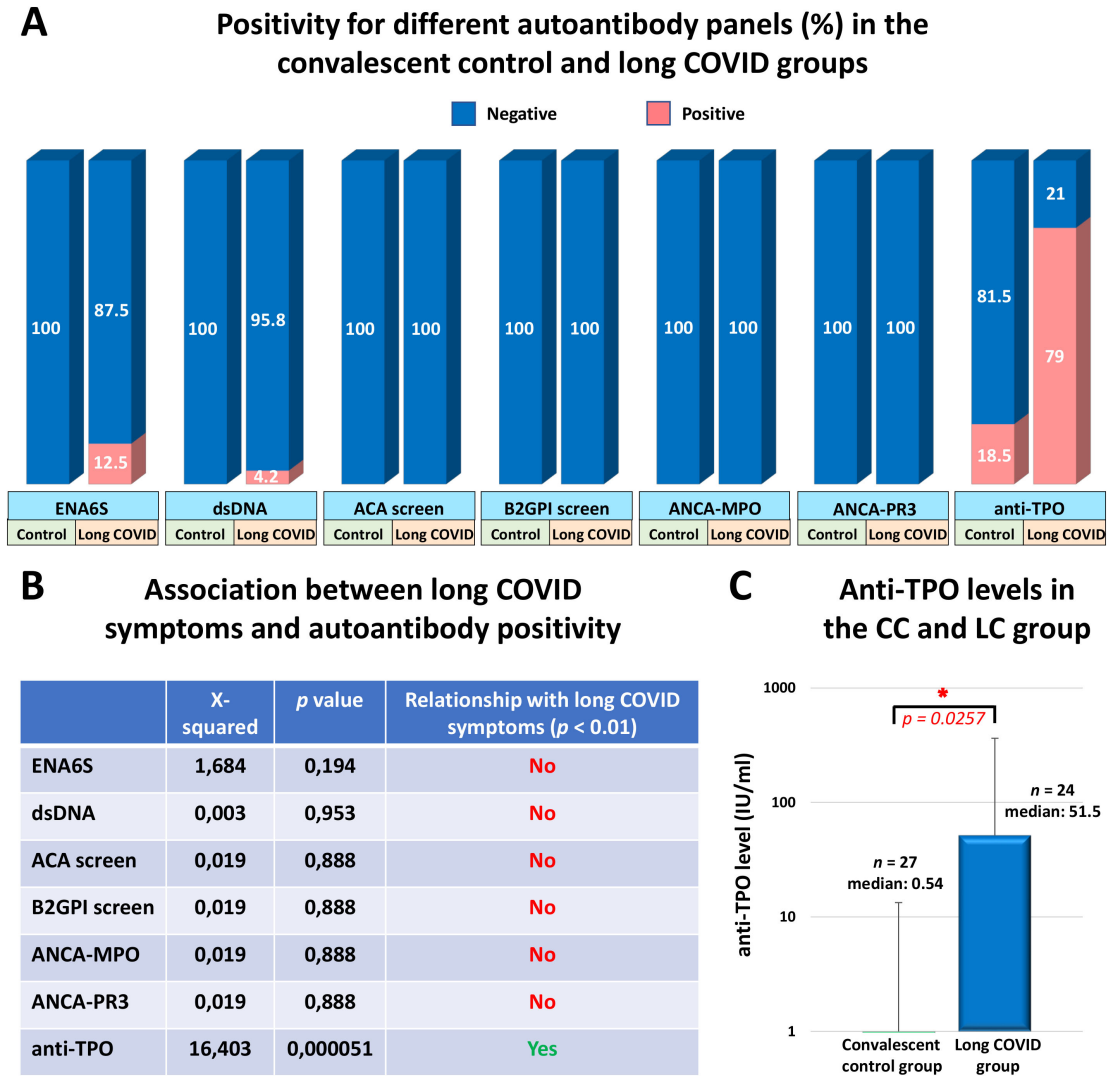
Serum positivity for distinct autoantibody panels in the convalescent control and long COVID groups (%), the association between long COVID symptoms and different autoantibody positivity, and serum anti-thyroid peroxidase (anti-TPO) antibody levels of study participants. **(A)** The positivity for different autoantibodies including six nuclear antigens within the ENA6S panel (SS-B, SS-A 52 kDa, Scl 70, Jo-1, snRNP/Sm, Sm, and SS-A 60 kDa), as well as anti-dsDNA, anti-cardiolipin, anti-β2-glycoprotein I, antineutrophil cytoplasmic antibodies, and anti-TPO was assessed with the ELISA and CMIA methods in the convalescent control and long COVID groups. **(B)** Pearson’s Chi-square test with Yates correction was used to test whether post-COVID symptoms and different AAB positivity are related, where p-values of <0.01 were considered statistically significant relationships. **(C)** Anti-TPO levels were determined in serum samples using a two-stage chemiluminescent microparticle immunoassay (CMIA) with the ARCHITECT Anti-TPO reagent kit (B2K47H). Statistical analysis utilized Student’s t-test, considering p-values of <0.05 as statistically significant (*). Box plots illustrate the median values with the interquartile range (lower and upper hinges) and ±1.5-fold of the interquartile range from the first and third quartiles (lower and upper whiskers).

**Figure 5:**
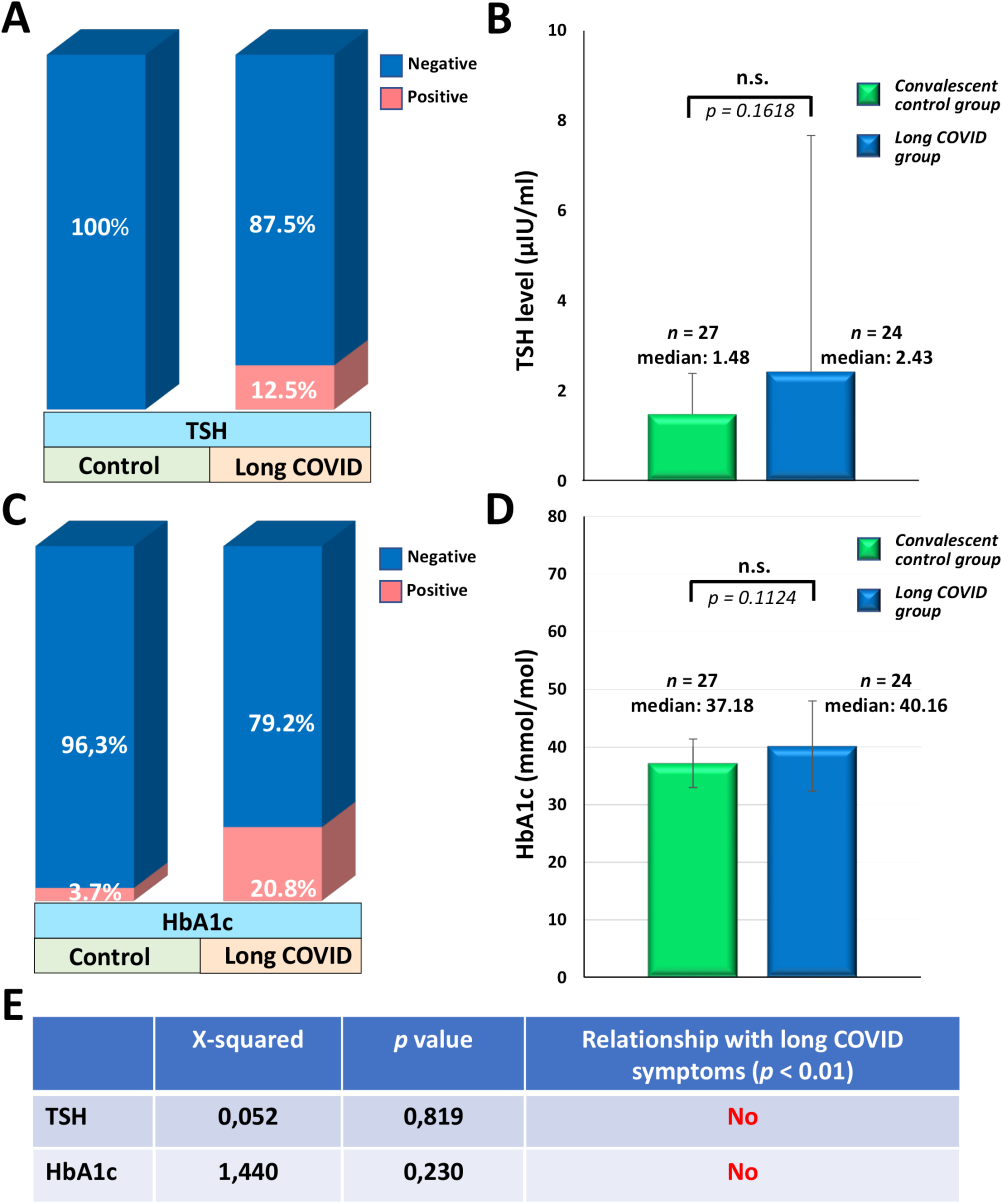
The prevalence of elevated TSH and HbA1c levels in the convalescent control and long COVID groups (%), the median TSH and HbA1c levels, and the relationship between long COVID symptoms and elevated levels of these parameters. **(A)** The ratio of elevated TSH levels (>4.94 µIU/ml) in the CC and LC groups was conducted according to the measurement of TSH levels. **(B)** Serum TSH levels of study participants were determined by a two-stage chemiluminescent microparticle immunoassay (CMIA) using the Architect TSH reagent kit (B7K62H). **(C)** The prevalence of elevated HbA1c levels (>42 mmol/mol) in the CC and LC groups was assessed according to the measurement of HbA1c levels. **(D)** HbA1c levels of the study participants were determined in EDTA-anticoagulated whole blood by the ADAMS A1c HA-8180V fully automated HPLC system. **(E)** The relationship between long COVID symptoms and elevated TSH or HbA1c levels was tested by Pearson’s Chi-square test with Yates correction, where p-values of <0.01 were considered statistically significant relationships. **(B, D)** Statistical analyses utilized Student’s t-test, considering p-values of >0.05 were considered non-significant (n.s.). Box plots illustrate the median values with the interquartile range (lower and upper hinges) and ±1.5-fold of the interquartile range from the first and third quartiles (lower and upper whiskers).

The quantitative determination of IgG antibodies against six different nuclear antigens, including SS-B, SS-A 52 kDa, Scl 70, Jo-1, snRNP/Sm, Sm, and SS-A 60 kDa, was performed using the ENA-6 S immunoenzymatic method. We found that the overall rate of IgG seropositivity was 0% (0/27) in the CC group and 12.5% (3/24) in the LC group. Only one long COVID patient (4.2%) showed anti-dsDNA IgG positivity, whereas none of the participants in the CC group exhibited increased levels of anti-dsDNA antibodies. Subsequently, serum samples of study participants were assayed for anti-cardiolipin (ACA screen; IgG, IgA, IgM), anti-beta2-glycoprotein I (BGPI screen; IgG, IgA, IgM), and anti-neutrophil cytoplasmic antibodies (ANCA-MPO, ANCA-PR3; IgG) by ELISA. Neither the long COVID patients nor the convalescent control participants showed elevated levels of these AABs. However, we observed dramatic differences in the prevalence of highly elevated anti-TPO antibodies between the two groups. The frequency of anti-TPO positivity (>5,61 IU/ml) in the CC group was 18.5% (5/27), whereas that in the LC group was 79% (19/24). The seropositivity rates for each AAB in the two groups are shown in **Figure 4A**. Based on Pearson’s chi-squared test, a strong statistical association was observed exclusively between the incidence of long COVID symptoms and anti-TPO seropositivity, as shown in **Figure 4B**. The median anti-TPO level in the LC group was substantially, approximately 95 times higher than that of the CC group, and this difference was statistically significant despite the considerable individual variability within the two groups (medians of 0.54 IU/ml in the CC and 51.5 IU/ml in the LC group; *p* = 0.0257) (**Figure 4C**). Due to the high prevalence of anti-TPO positivity, which reached 79% in the LC group, we measured serum TSH levels and compared them with those of the CC group. In the LC group, only 3 out of 24 individuals (12.5%) showed elevated TSH levels (>4.94 µIU/ml), while all participants in the CC group had normal TSH levels within the reference range (**Figure 5A**). Additionally, we did not find a statistically significant difference in TSH levels between the CC and LC groups (medians of 1.48 µIU/ml in the CC and 2.43 µIU/ml in the LC group; *p* = 0.1618) (**Figure 5B**). To determine whether these participants had already been anti-TPO positive before their SARS-CoV-2 infection, we reviewed historical data from their medical records. Among the anti-TPO-positive participants in the LC group, 58% (11/19) had already been anti-TPO-positive before the COVID-19 pandemic. However, 42% (8/19) had no recorded anti-TPO measurements in their medical histories, as their TSH levels were within the reference range during examinations conducted in 2018 and 2019. Since anti-TPO positivity can occur even with normal TSH levels, as observed in 16 of the 19 long COVID participants, it cannot be definitively concluded that these participants were negative before their acute infection. Similarly, 60% (3/5) of the anti-TPO-positive participants in the CC group were also positive before the pandemic. Finally, to investigate the relationship between diabetes and long COVID, we measured the HbA1c levels of the study participants. In the CC group, only 1 out of 27 individuals (3.7%) had elevated HbA1c levels (>42 mmol/mol), whereas 5 out of 24 individuals (20.8%) showed high levels in the LC group (**Figure 5C**). However, no statistically significant difference was found between the two groups (medians of 37.18 mmol/mol in the CC group and 40.16 mmol/mol in the LC group; *p* = 0.1124) (**Figure 5D**). Accordingly, based on Pearson’s chi-square test, no relationship was found between long COVID symptoms and elevated TSH or HbA1c levels among our study participants (**Figure 5E**). For participants with elevated HbA1c levels, no clinical data from the pre-COVID-19 period suggest the presence of diabetes, they had neither been investigated specifically for diabetes nor diagnosed with it. Nevertheless, it cannot be excluded that these participants had undiagnosed diabetes before the COVID-19 pandemic. Therefore, we cannot conclude that their cases represent newly developed diabetes associated with COVID-19.

## Discussion

The healthcare workers in this study had all experienced SARS-CoV-2 infection, predominantly confirmed by a PCR test. The 51 participants were classified into two groups: individuals in the convalescent control group reported no persistent symptoms after recovery. In contrast, all participants in the long COVID group reported persistent or recurring symptoms lasting at least three months. 50 out of the 51 participants were female, with an average age of 49 years (CC group) and 52 years (LC group) at the time of study. All participants received the first and second vaccine doses 21 days apart between January and March 2021. Subsequently, between September and November 2021, an average of 7.4 months after the second dose, booster vaccination was administered. 86% of the CC group and 96% of the LC group received three doses of mRNA-based BNT162b2 vaccine. The remaining participants were immunized with a heterologous vaccination regimen, which included the Ad26.COV2.S, Gam-COVID-Vac, or BBIBP-CorV vaccines, in addition to BNT162b2. Our measurements were conducted on average 21 months after booster vaccination, between June and August 2023. There were significant differences between the two groups regarding the severity and frequency of acute symptoms as well as the time of infection relative to the individual vaccine doses. Long COVID patients reported acute symptoms such as general weakness, cough, headache, taste and smell disorders, high fever, muscle, and joint pain more frequently and with greater severity than convalescent control participants. Post-acute symptoms persisted for an average of 3 months in 54%, 6 months in 25%, and more than 6 months in 21% of patients with long COVID. Notably, while 82% of participants in the CC group became ill after receiving the third dose, with only 9% being infected before the first dose, 50% of long COVID patients were infected before receiving the first dose, and only 45% after receiving the booster dose. Therefore, the average time elapsed since confirmed SARS-CoV-2 infection was 18.2 months in the CC group and 23.1 months in the LC group at the time of investigation. This result supports the growing consensus that receiving multiple doses of the COVID vaccine before the first SARS-CoV-2 infection can significantly reduce the risk of long-term symptoms (41–43).

Recently, several mechanisms have been associated with long COVID, such as immune dysregulation, activation or dysfunction of the vascular endothelium, mast cell activation, autoimmunity, neurological signaling dysfunction, dysbiosis, and reactivation of latent viruses, and an increasing amount of evidence supports that these abnormalities are associated with the development of persistent virus reservoirs (5, 6). The undebatable evidence for the existence of virus reservoirs is the identification of SARS-CoV-2-specific proteins or RNA in tissue biopsies or serum samples, as demonstrated in several studies involving long COVID patients (12–16). However, we can also deduce the existence of these virus reservoirs from T cell-mediated and humoral immune responses. Our results showed that almost two years after the acute infection, a significantly higher T cell response was observed in the LC group than in the CC group against the spike (*p* = 0.0086), nucleocapsid (*p* = 0.0272), and membrane (*p* = 0.0120) proteins. However, we observed almost identical T cell responses in the two groups against those SARS-CoV-2 epitopes that show a high degree of homology with endemic coronavirus strains. Additionally, considerably higher levels of anti-S IgG serum antibodies were measured in the LC group; however, this difference was not statistically significant (*p =* 0.3147). These results align with the findings of other groups, as a significantly higher number of circulating SARS-CoV-2-specific T cells were reported in long COVID patients than in convalescent control individuals (17, 18). Additionally, higher SARS-CoV-2-specific serum antibody levels were measured in patients with long COVID than in control individuals (19, 36). However, contrary results are also present in the literature. It was reported that no considerable differences were observed in antibody and T cell responses between long COVID patients and convalescent control individuals 18 weeks after acute infection (20). Another study reported lower anti-S1/S2 and neutralizing antibody levels in the longer term, one year after acute COVID-19, along with a similar number of SARS-CoV-2-reactive peripheral T cells compared to the control group (21).

Based on our current knowledge, autoimmune reactions during acute COVID-19 are likely consequences of virus-induced immune activation and inflammatory cascades rather than a direct effect of the virus. There is ample evidence that acute COVID-19 can induce a large and diverse autoantibody repertoire, affecting numerous self-antigens in the heart, intestines, lungs, kidneys, brain, skin, skeletal muscle, and many other organs and tissues (22–26). Among these antibody targets, we can find the members of the type I interferon pathway (44, 45), G-protein coupled receptors and the renin-angiotensin system (46, 47), antigens of the central and peripheral nervous system (22, 48), dsDNA (49), myelin associated glycoprotein (25), pulmonary surfactant protein A1 (25), prothrombotic anti-phospholipids and phospholipid-binding proteins such as cardiolipin, β2-glycoprotein I, prothrombin, phosphatidylserine (33–35, 50, 51), nuclear antigens (27, 31), neutrophil cytoplasmic antigens (52, 53), thyroid peroxidase (54, 55), among many others. Although the virus can induce an extremely wide spectrum of AABs, so far only a few studies have found a clear association with long COVID. AABs specifically identified in patients with long COVID, but not during acute COVID-19, target several antigens. These include receptors of the GPCR signaling pathway (36, 37), which are associated with the immunopathogenesis of cardiological and neurological autoimmunity, including dysautonomia and POTS; nuclear antigens (27), which are linked to rheumatological symptoms, lupus, and vasculitis; CCP and TG (38), associated with rheumatoid arthritis and coeliac disease, respectively; DSG2 (39), related to cardiac pathology; and sex-specific antigens (40), which are connected to a wide range of long COVID symptoms. During our analyses, we compared the LC and CC groups regarding AABs that had been previously identified in patients with acute disease: anti-dsDNA, anti-cardiolipin, anti-β2-glycoprotein I, anti-neutrophil cytoplasmic antibodies, and anti-TPO. Additionally, we analyzed the levels of anti-nuclear antibodies that have been previously associated with long COVID as well. It is important to emphasize that none of the participants had COVID-19 at the time of the investigation, and the average time since the last confirmed SARS-CoV-2 infection was 18.2 months in the CC group and 23.1 months in the LC group. In the case of different nuclear antigens, including SS-B, SS-A 52 kDa, Scl 70, Jo-1, snRNP/Sm, Sm, and SS-A 60 kDa, the overall rate of IgG seropositivity was 0% (0/27) in the CC group and 12.5% (3/24) in the LC group. The frequency of anti-dsDNA positivity in the CC group was 0% (0/27), whereas that in the LC group was only 4.2% (1/24). Regarding anti-cardiolipin, anti-β2-glycoprotein I, and antineutrophil cytoplasmic antibodies, we did not detect elevated serum Ig levels in any of the participants in the CC and LC groups. Conversely, we found a significant difference in anti-thyroid peroxidase IgG positivity, as the frequency of highly elevated anti-TPO antibodies in the CC group was 18.5% (5/27), whereas in the LC group, it was 79% (19/24). According to Pearson’s chi-squared tests, we found a statistical association between long COVID symptoms and elevated serum AAB levels exclusively in the case of anti-TPO. However, only 3 out of 24 individuals had elevated serum TSH levels in the LC group (12.5%), while in the CC group, it was within the reference range for all participants. To understand the causal relationship between anti-TPO positivity and post-COVID syndrome, we reviewed the participants’ medical history. Among the 19 anti-TPO-positive participants in the LC group, 11 (58%) had already been anti-TPO positive before the onset of SARS-CoV-2, while in 8 out of 19 participants (42%), no anti-TPO testing had been conducted before the COVID-19 pandemic, as their TSH levels were within the reference range. However, we cannot conclusively state that these participants were anti-TPO negative before COVID-19, and AAB induction is a consequence of SARS-CoV-2 infection since anti-TPO positivity can occur without hypothyroidism, as observed in 16 out of 19 participants in the long COVID group. A similar pattern was found in the CC group, where 60% (3/5) of anti-TPO-positive participants had already been positive before the pandemic. Several case reports have shown that, in some individuals, the anti-TPO titer increases during COVID-19 and the post-recovery period. A study involving 124 hospitalized COVID-19 patients reported that 94.4% of survivors were euthyroid 6 months after hospital discharge, whereas, in certain patients, the period following recovery was linked to notably higher anti-TPO titers and the emergence or persistence of subclinical hypothyroidism (55). Another large prospective study showed that the prevalence of autoimmune thyroid disease in COVID-19 survivors was double that in the control group. The prevalence of TPO antibodies 3 months after acute infection was 15.7% in COVID-19 survivors compared to 7.7% in age- and sex-matched controls, suggesting a role for COVID-19 in eliciting thyroid autoimmunity (54). Both groups recommended the need for monitoring the development of thyroid dysfunction and autoimmunity after COVID-19. In light of these results, our finding that highly elevated anti-TPO titers were measured in 79% of the long COVID patients compared to the 18.5% prevalence in the CC group, even 23.1 and 18.2 months after COVID-19, respectively, strongly suggests a clear association between highly elevated anti-TPO titers and long COVID.

A persistent virus reservoir providing continuous antigenic stimulation and maintaining a chronic, low-grade inflammatory and immunologic state can constantly trigger diabetes through several autoimmune mechanisms, including epitope spreading, molecular mimicry, and bystander activation (56–58). For this reason, we finally evaluated the incidence of diabetes and examined its relationship with long COVID by measuring HbA1c levels among the study participants. We observed elevated HbA1c levels in 1 of 27 individuals (3.7%) in the CC group and 5 of 24 individuals (20.8%) in the LC group. The difference between the two groups regarding the HbA1c levels was not statistically significant, and Pearson’s chi-squared test did not reveal an etiological link between diabetes and long COVID twenty-three months after the acute infection. For participants with elevated HbA1c levels, their medical history before the COVID-19 pandemic does not indicate any data suggesting diabetes. They had neither been investigated specifically for diabetes nor diagnosed with it. Nonetheless, we cannot conclude that these cases represent new-onset diabetes as a consequence of COVID-19, as it remains possible that these participants had undiagnosed diabetes before the COVID-19 pandemic. Regarding this issue, a systematic review revealed that 44% of the studies related to the link between diabetes and post-COVID syndrome identified diabetes as a significant risk factor for long COVID, with raised relative risk ranging from 7% to 342%. In contrast, 56% of these studies did not suggest diabetes as a risk factor for the development of long COVID. In addition, 86% of the studies reporting new-onset diabetes found that COVID-19 was significantly associated with newly developed diabetes, with raised relative risks ranging from 11% to 276% (59).

## Conclusions and limitations

Our findings confirm that those who contracted COVID-19 before vaccination were more likely to develop long COVID than those vaccinated before their first infection. Moreover, among patients with long COVID, prior SARS-CoV-2 infection generally led to a greater number of symptoms with more severity compared to those individuals who did not experience long-term symptoms. Importantly, 21 months after the booster vaccination, the long COVID group exhibited a significantly higher T cell response against spike, nucleocapsid, and membrane proteins, along with moderately elevated anti-S IgG serum antibody levels compared to the convalescent control group. Additionally, we observed a considerably higher rate of anti-TPO positivity in the long COVID group than in the convalescent control group (79 vs. 18.5%) suggesting that highly elevated anti-TPO titers and post-COVID syndrome are related. However, our findings do not support the conclusion that elevated anti-TPO levels (0.54 vs. 51.5 IU/ml) are a direct consequence of COVID-19, as 58% of the long COVID patients who tested positive for anti-TPO almost two years after COVID-19 were already positive before their acute infection. For the remaining participants, anti-TPO levels were not evaluated before COVID-19 leaving open the possibility of elevated titers despite normal TSH levels. Furthermore, we more frequently measured elevated anti-nuclear antibody and HbA1c levels among patients with long COVID than in the case of control participants, but there was no statistically significant difference between the two groups. In contrast, we did not detect elevated levels of anti-cardiolipin, anti-β2-glycoprotein I, or anti-neutrophil cytoplasmic antibodies in any of the study participants. The main limitation of our work is the small sample size, as only 51 healthcare workers could be recruited for the study, who met the inclusion criteria: 27 individuals in the convalescent control group and 24 in the long COVID group. Another limitation of the study is that we only identified SARS-CoV-2-responsive IFN-γ secreting T cells without investigating the different T cell subsets.

## Data Availability

The original contributions presented in the study are included in the article/**Supplementary Material**. Further inquiries can be directed to the corresponding author.
