# Supplementary material for: High prevalence of long COVID in anti-TPO positive euthyroid individuals with strongly elevated SARS-CoV-2-specific T cell responses and moderately raised anti-spike IgG levels 23 months post-infection
Source: Front Immunol. 2024 Oct 10;15:1448659. doi: 10.3389/fimmu.2024.1448659 (PMC11499158; doi:10.3389/fimmu.2024.1448659)
Supplement: Supplementary file 1 [file DataSheet1.docx]

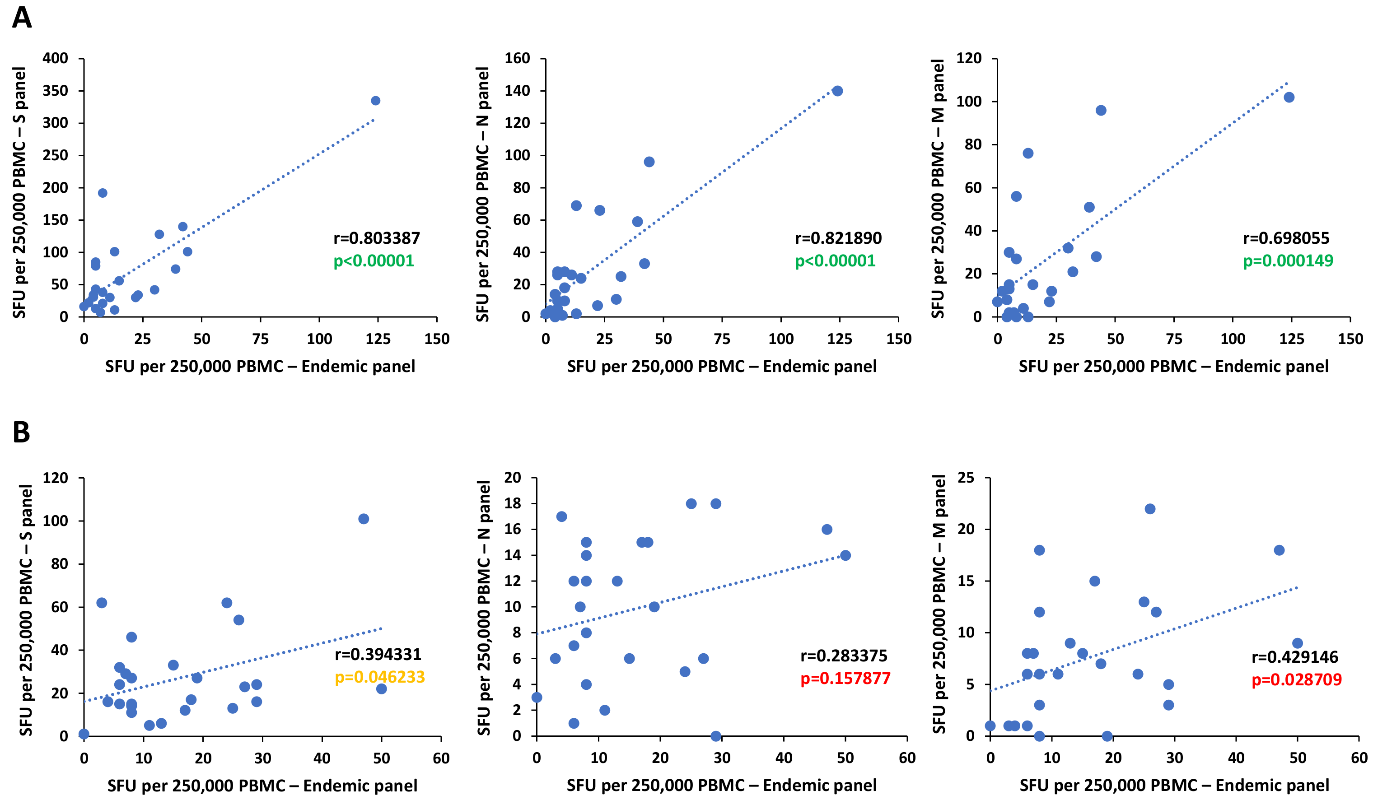


**Supplementary Figure S1.** Pearson’s correlation analysis between T cell responses against individual structural SARS-CoV-2 viral proteins (spike, nucleocapsid, membrane) and T cell responses against epitopes characteristic of endemic coronavirus strains in the long COVID (A) and convalencent control (B) groups.
